# Dungeons & Dragons in Schools: An Underexplored Opportunity for Early Anxiety Support

**DOI:** 10.3390/bs16101795

**Published:** 2026-10-01

**Authors:** Brad Hatton, Marie House

**Affiliations:** 1 School of Psychology, University of Nottingham, University Park, Nottingham NG7 2RD, UK; brad.hatton@nottingham.ac.uk; 2 Nottinghamshire Educational Psychology Service, Mansfield NG18 2TB, UK

**Keywords:** Dungeons & Dragons, tabletop role-playing games, adolescent anxiety, early support, schools, narrative approaches, social connectedness, psychological wellbeing

## Abstract

Adolescent anxiety presents a growing challenge for schools, with emotional difficulties affecting educational engagement, attendance and wellbeing before some pupils meet thresholds for specialist mental health support. This creates a need to investigate accessible and non-stigmatising forms of early support within schools. Emerging TTRPG research has described experiences involving social connection, confidence, self-expression and emotional exploration, but direct evidence concerning secondary-aged pupils experiencing emerging or subthreshold anxiety remains limited. This opinion paper brings together findings from existing TTRPG research, mechanisms identified in broader psychological theory and related narrative approaches, and propositions concerning how these may operate through school-based TTRPG participation. Its contribution is a conceptual model linking components of TTRPG participation with five overlapping proposed mechanisms: narrative distance; psychological safety and social connectedness; intrinsic motivation and agency; identity exploration and self-efficacy; and cognitive flexibility and emotional regulation. The paper also outlines an illustrative school-based delivery model and a staged agenda for future research. These pathways are theoretically plausible rather than empirically established, and TTRPG participation should not be assumed to reduce anxiety or replace specialist assessment or treatment. Initial research should establish feasibility, acceptability and safety, including potential adverse experiences, before psychological or educational outcomes are evaluated.

## 1. Introduction

Anxiety is one of the most common mental health difficulties in adolescence. National data indicate that rates of probable mental disorder rose between 2017 and 2020 and remained stable at elevated levels between 2022 and 2023 (NHS England, 2023), within a wider cost-of-living context associated with financial stress and poorer mental health across the UK population (Mental Health Foundation, 2023). Schools are expected to provide early support in the context of long waiting lists and limited access to specialist services. However, many young people experience emerging or subthreshold anxiety that, while not meeting diagnostic criteria, continues to affect learning, participation, and overall wellbeing. This paper focuses specifically on secondary-aged pupils experiencing emerging or subthreshold anxiety that affects their participation or engagement in school. The proposed approach is not intended to replace specialist assessment or evidence-based clinical treatment where these are indicated.

These challenges arise within a wider context of rising school absence, with persistent absence across the autumn and spring terms increasing from 10.5% in 2018–2019 to 17.6% in 2024–2025 (Department for Education, 2025). Within Connolly et al.’s (2023) parent-report sample, 94.3% of school attendance difficulties were reported as partially or fully emotionally based, and early support at the onset of attendance concerns is therefore considered important (Education Endowment Foundation, 2025). School attendance difficulties are not synonymous with anxiety, but these findings illustrate one context in which emotional distress may affect participation in education.

This creates a case for investigating accessible, non-stigmatising forms of support that can respond to young people’s interests and motivations within school settings. Therapeutic story writing provides one relevant precedent, using metaphor and narrative distance to support emotional expression and processing and enabling pupils to explore difficult experiences through symbolic characters or plots (Waters, 2004). Tabletop role-playing games (TTRPGs), including Dungeons & Dragons, may extend these principles through collaborative and interactive narratives in which participants contribute to the development of characters, events, and shared stories.

Small-scale studies and practice literature concerning TTRPGs, drawn largely from adult, clinical, or community contexts, have described experiences relating to confidence, identity exploration, emotional expression, and social connection (Yuliawati et al., 2024; Connell, 2023). Features of TTRPG participation may also create opportunities for shared problem-solving, fictionalised failure, and the rehearsal of coping strategies within a narrative frame. Although the relevance of this evidence to secondary-school pupils experiencing anxiety has yet to be established, it identifies processes warranting further investigation.

Evidence from child and youth mental health services suggests that flexible, person-centred approaches responsive to young people’s needs and preferences may support engagement and increase their involvement in decisions about their care (Gondek et al., 2017). Broader motivational theories similarly propose that activities balancing challenge with enjoyment and personal investment can support engagement and experiences of competence (Csikszentmihalyi, 1990), while opportunities for choice and participation may support autonomy and intrinsic motivation (Ryan & Deci, 2000). Applied to TTRPGs, these bodies of work provide a theoretical rationale for examining whether collaborative storytelling, meaningful choice, and participation in a shared activity might help sustain engagement in school-based support.

The feasibility and sustainability of any new school-based approach must also be considered. The adaptable pace, content, and complexity of TTRPGs could potentially support their use with different groups, while collaborative storytelling allows participants to influence the direction of the activity. The practical significance of this flexibility within schools will depend on resource requirements, facilitator knowledge and training, safeguarding, management of group dynamics, and the organisational conditions necessary for safe and sustainable implementation.

Broader longitudinal evidence also identifies social connectedness as a protective factor in adolescence. Having both strong school and social connectedness in early secondary school is associated with better later outcomes, while low school connectedness is associated with greater later anxiety/depressive symptoms (Bond et al., 2007). Although this evidence is not specific to TTRPG participation, it supports examining social connection as a possible pathway within a school-based TTRPG approach.

Despite growing academic interest in tabletop role-playing games and their possible applications within wellbeing and mental health research (Martinolli, 2025), their potential role in supporting adolescents experiencing emerging anxiety in secondary schools remains underexplored. There is particularly limited understanding of how features of collaborative role-play, including narrative distance, shared storytelling, agency, identity exploration, and social interaction, might operate within school-based support. The central proposition considered here is that positioning young people as active contributors to a shared fictional narrative may enable engagement with emotionally relevant themes in ways that feel indirect, participatory, and personally meaningful. The extent to which these processes translate into improved wellbeing or anxiety-related outcomes requires empirical investigation.

This opinion paper examines TTRPGs as a possible approach to early anxiety support in secondary schools. It separates findings from the existing TTRPG literature from mechanisms drawn from broader psychological theory and from propositions that still require empirical testing. A conceptual model links features of TTRPG participation with proposed mechanisms and outcomes, before consideration is given to what a theoretically informed school-based approach could involve. The paper then identifies priorities for evaluating feasibility, acceptability, adverse effects and any contribution to early anxiety support.

## 2. Conceptual Framework

### 2.1. Overview

Tabletop role-playing games have increasingly been explored both as naturally occurring recreational activities and as intentionally structured forms of support. Small-scale TTRPG studies and practice literature have described experiences relating to social connection, confidence, interpersonal interaction, self-expression, and emotional exploration (Connell, 2023; Yuliawati et al., 2024). However, direct evidence concerning their use with secondary-aged pupils experiencing emerging anxiety remains limited.

The framework presented here therefore draws on three related but distinct forms of evidence: findings reported in existing TTRPG research; mechanisms identified in broader psychological theory and related narrative approaches; and propositions concerning how those mechanisms might operate within school-based TTRPG participation. Within this framework, a proposed mechanism should not be interpreted as an empirically established process of change. Rather, it identifies a theoretically plausible pathway that can inform the design and evaluation of future school-based research.

Five overlapping mechanisms are considered: narrative distance; psychological safety and social connectedness; intrinsic motivation and agency; identity exploration and self-efficacy; and cognitive flexibility and emotional regulation. Their operation is unlikely to be inherent to all forms of role-playing or to occur automatically through participation. The extent to which they are supported will depend on features such as the game system, narrative structure, facilitator practice, group composition, and developmental appropriateness of the activity.

### 2.2. Narrative Distance

Therapeutic story writing uses metaphor and narrative distance to support emotional exploration, expression, and meaning-making in school contexts (Waters, 2004). Narrative distance refers to engagement with emotionally significant experiences through fictional characters or scenarios, allowing difficult themes to be approached without requiring direct personal disclosure. The fictional frame can create separation between the individual and the subject being explored while retaining sufficient emotional relevance for reflection.

TTRPGs may extend this process by locating fictional characters within an unfolding collaborative narrative. Players make decisions, encounter challenges, and experience consequences through characters who are connected to, but distinct from, their everyday identities. This may enable emotional themes to emerge through events within the story rather than through direct questioning or discussion of personal experiences.

Existing TTRPG studies have reported experiences involving social connection, confidence, challenging interpersonal situations, and collaborative problem-solving (Abbott et al., 2022; Kilmer et al., 2024). Although these studies do not isolate narrative distance as the mechanism producing those experiences, they illustrate how fictional role-play can combine indirect exploration with social participation. Within the present framework, narrative distance is therefore proposed as one pathway through which young people might engage with emotionally relevant material while retaining control over how closely it is connected to their own experiences.

### 2.3. Psychological Safety and Social Connectedness

School belonging and social connectedness are important to young people’s mental wellbeing (Bond et al., 2007). School belonging refers to the extent to which pupils feel accepted, respected, included, and supported within their school environment (Allen et al., 2018). This may be particularly important for young people experiencing anxiety, as difficulties relating to social, academic, and personal functioning can restrict participation in school activities and heighten concerns about judgement or failure (Spence, 1998).

Emerging TTRPG research suggests that collaborative play can support social connection and confidence while providing opportunities to practise challenging interpersonal situations and shared problem-solving (Abbott et al., 2022; Kilmer et al., 2024). TTRPG groups typically work towards shared narrative goals, requiring participants to exchange ideas, respond to one another, negotiate decisions, and contribute to collective outcomes. These features may create repeated opportunities for positive peer interaction and a developing sense of group membership.

Psychological safety should not, however, be treated as an automatic property of fictional or game-based activity. Role-play may still involve social exposure, uncertainty, disagreement, or fear of making mistakes in front of others. Its development is likely to depend on clear expectations, supportive facilitation, respectful group norms, and meaningful participant choice. Where these conditions are established, the combination of a fictional frame and shared group purpose may reduce the perceived personal risk associated with contributing ideas, experimenting with responses, or making mistakes.

### 2.4. Intrinsic Motivation, Agency and Engagement

Self-Determination Theory proposes that intrinsic motivation is supported when activities meet needs for autonomy, competence, and relatedness (Ryan & Deci, 2000). Within educational settings, agentic engagement describes the ways in which learners actively contribute to and influence their learning environment, including by expressing preferences and shaping the direction of an activity. This form of engagement has been associated with achievement and the development of more autonomy-supportive learning environments (Reeve, 2013).

These perspectives offer a theoretical basis for considering how TTRPG participation might support engagement. Although a facilitator establishes the narrative context and boundaries of play, participants make meaningful choices, contribute ideas, develop characters, and influence how the shared story unfolds. Autonomy may be supported through these choices, competence through increasingly successful participation and problem-solving, and relatedness through collaboration with other group members.

The co-constructed nature of the story is particularly important. Participants are not simply completing activities selected by an adult; their decisions become part of the evolving narrative and influence what happens next. This may create a stronger sense of ownership and personal investment than participation alone. For pupils experiencing emerging anxiety, such involvement could make support feel less externally imposed and provide reasons to continue participating beyond an explicitly therapeutic goal. Whether these features improve the acceptability or sustained engagement of school-based support requires direct investigation.

### 2.5. Identity Exploration and Self-Efficacy

Adolescence is an important period of identity development in which young people explore and shape their sense of self through social interaction and experience (Erikson, 1968). Anxiety may restrict opportunities for this exploration where concerns about judgement, failure, or uncertainty make unfamiliar social roles and activities difficult to approach.

Narrative identity theory proposes that people make sense of themselves through the construction of evolving personal stories (McAdams, 2001). Fictional characters may provide a means of experimenting with qualities, values, relationships, and possible ways of acting while maintaining some separation from the everyday self. In a TTRPG, this exploration occurs within a responsive social environment: character choices shape the narrative and receive reactions from other participants.

These experiences may also relate to self-efficacy, understood as belief in one’s capacity to organise and carry out the actions needed to achieve particular outcomes (Bandura, 1997). Through their characters, participants may encounter manageable challenges, contribute to group success, recover from setbacks, and see the consequences of their decisions. Such experiences could create opportunities to explore competence and agency within the fictional setting.

Dungeons & Dragons provides a particularly structured example through character creation, collaborative storytelling, rule-governed challenges, and continuing character development. Participants can try unfamiliar roles or behaviours while attributing those choices to a fictional character. The extent to which confidence or efficacy experienced through a character transfers beyond the game is likely to depend on opportunities to recognise, reflect upon, and connect these experiences with situations outside the narrative. Identity exploration and self-efficacy are therefore treated here as related proposed pathways rather than assumed outcomes of participation.

### 2.6. Cognitive Flexibility and Emotional Regulation

Cognitive flexibility and emotional regulation are relevant to young people’s capacity to respond to challenge and uncertainty. Anxiety is often associated with rigid thinking, heightened threat perception, and avoidance, which can limit responses to unfamiliar or unpredictable situations (Spence, 1998). Cognitive flexibility refers to the capacity to shift perspective and adapt thinking in response to changing demands and forms part of broader executive functioning processes (Diamond, 2013). Emotional regulation refers to the processes through which individuals influence which emotions they have, when they have them, and how they experience and express them (Gross, 1998).

TTRPG participation requires players to respond to developing scenarios, consider incomplete information, adapt plans when outcomes are unexpected, and take account of perspectives other than their own. Rules and fictional boundaries provide structure, while dice rolls, group decisions, and narrative developments introduce uncertainty. These features may offer opportunities to practise changing strategies and tolerating outcomes that cannot be fully controlled.

Gameplay can also evoke emotions connected with anticipation, frustration, disappointment, excitement, and success within a contained fictional context. Supportive facilitation may help participants remain engaged following setbacks, consider alternative responses, and reflect on how characters manage emotionally challenging situations. The framework therefore treats cognitive flexibility and emotional regulation as capacities that TTRPG participation may allow young people to rehearse. Whether repeated practice produces changes beyond the game, including changes in anxiety-related functioning, remains to be established through empirical research.

### 2.7. Proposed Conceptual Model

The mechanisms described above are unlikely to operate as isolated processes. A single moment of gameplay may involve narrative distance, social connection, agency, identity exploration, flexible thinking, and emotional experience simultaneously. For example, a pupil navigating a challenge through their character may contribute to a group decision, receive positive feedback from peers, adapt to an unexpected outcome, and experience a sense of competence within the same narrative sequence.

The potential value of TTRPGs may therefore lie in the interaction between their components rather than in any single game feature. Table 1 summarises the proposed relationships between central components of TTRPG participation, the mechanisms through which they may operate, proximal experiences that could be observed during participation, and the outcomes that future research would need to evaluate.

This model places proximal experiences, such as participation, perceived safety, social interaction, and willingness to return, between game components and longer-term psychological outcomes. It therefore does not assume that taking part in a TTRPG directly reduces anxiety. Instead, it proposes pathways through which particular forms of carefully structured participation might contribute to experiences relevant to early anxiety support.

The model also highlights the importance of implementation. The same game component may operate differently depending on the facilitator, group dynamics, level of participant choice, narrative content, and accessibility of the rules. These conditions must therefore be examined alongside mechanisms and outcomes rather than treating the game system itself as the intervention. This provides a conceptual basis for considering what a school-based TTRPG approach might look like in practice and for developing testable questions for future feasibility and acceptability research.

## 3. Considerations for School-Based Development and Implementation

The conceptual framework identifies several pathways through which carefully structured TTRPG participation might contribute to experiences relevant to early anxiety support. Translating this framework into a school-based approach would require decisions about the intended population, programme structure, game system, facilitation and implementation conditions. The following considerations draw both on emerging TTRPG research and on broader evidence concerning young people’s experiences of school-based mental-health interventions. Evidence from the latter does not establish the effects of TTRPG participation, but can identify conditions likely to influence the accessibility, acceptability and feasibility of any future school-based programme.

### 3.1. Intended Population and Boundaries of Support

The approach considered in this paper is intended primarily for secondary-aged pupils experiencing emerging or subthreshold anxiety-related difficulties that affect their participation, engagement or wellbeing in school. These difficulties might include avoidance of unfamiliar activities, reduced classroom participation, concerns about social judgement or growing disengagement from school. The proposed target population is therefore narrower than pupils requiring general emotional wellbeing or attendance support. Attendance difficulties may be one indication of anxiety-related need, but are not a sufficient criterion for participation, while reluctance to engage with traditional interventions may influence the acceptability of the approach without itself indicating inclusion.

Identification would need to take place through schools’ existing systems of early support, informed by discussion with the young person and, where appropriate, their parents or carers and relevant professionals. Previous school-based intervention research suggests that pupils can feel confused, judged or reluctant when selected by adults without understanding why support has been offered. However, reliance upon self-referral alone may exclude young people who lack confidence, do not recognise their difficulties or feel uncertain about requesting help (McGeechan et al., 2019; McKeague et al., 2018). A supported-choice model may therefore be appropriate, in which trusted adults explain the activity clearly and encourage participation while preserving the young person’s ability to decline.

Suitability should be considered separately from level of need. Previous experience of Dungeons & Dragons should not be required, but participants would need to express some willingness to try collaborative imaginative play. The proposed approach is not intended to replace specialist assessment or evidence-based treatment where these are required, and participation should not delay referral to appropriate services. Where a young person is already receiving specialist support, involvement in a TTRPG group would need to be considered in relation to that wider plan rather than presented as an alternative to it.

### 3.2. An Illustrative School-Based Delivery Model

As no validated school-based TTRPG programme for emerging adolescent anxiety has yet been established, any proposed structure should be treated as a prototype for feasibility testing rather than a prescribed intervention model. One possible starting point would be a group of four to six pupils meeting weekly for approximately 45–60 min over six weeks. These parameters may be compatible with some existing pastoral or targeted-support arrangements, but their acceptability and practicality would need to be examined across different schools and pupil groups.

An initial “Session Zero” could introduce the activity, establish expectations and allow pupils to influence the form of the shared story. This would include discussing how the game works, identifying accessibility needs, agreeing boundaries for narrative content and explaining how participants can pause or step back from an activity. Character creation could be undertaken collaboratively, using simplified or pre-generated options where helpful. This session would also provide an opportunity for the facilitator to observe how pupils respond to the format and whether the proposed group composition appears workable.

Subsequent sessions could follow a broadly consistent structure. A brief opening check-in and narrative recap might be followed by approximately 30–40 min of collaborative gameplay, concluding with a short check-out concerning the experience of the session and any preferences for future play. Reflection could remain primarily within the fictional frame, for example by considering what a character noticed, which options the group considered or how the party responded to an unexpected outcome. Pupils would not be required to explain how fictional events relate to their own lives, although personally meaningful reflection could be acknowledged where initiated by the young person.

The co-constructed narrative is central to the proposed approach. Participants would not simply complete a sequence of challenges selected by an adult; their decisions, ideas and character actions would materially influence subsequent events. The facilitator could use open narrative questions and situations involving uncertainty, collaboration or competing possibilities while avoiding predetermined “correct” responses. This preserves opportunities for agency and creative participation and distinguishes the proposed model from using a commercially available adventure without adaptation.

Although enjoyment may support participation, gameplay would still require a coherent rationale. Research concerning other school-based interventions suggests that young people value active and varied activities but may disengage when the connection between an activity and its purpose is unclear (Neill et al., 2023). The purpose and boundaries of the group should therefore be explained in accessible language, while avoiding an expectation that every moment of gameplay must become an explicit therapeutic exercise.

### 3.3. Game-System Selection and Adaptation

Dungeons & Dragons provides a recognisable example through which the proposed framework can be explored. Character roles and abilities offer structure, continuing character development makes progression visible, and the combination of rules, collaborative decision-making and uncertain outcomes can support an unfolding shared narrative. Its fantasy setting also provides scope for metaphor and for approaching emotionally relevant themes through fictional events (Baker et al., 2023; Henrich & Worthington, 2023).

These characteristics are not unique to Dungeons & Dragons, and the available evidence does not demonstrate that it is superior to other TTRPG systems. Its rules can involve substantial cognitive, literacy and numeracy demands, while its emphasis on fantasy and combat and its wider cultural associations may be unappealing or uncomfortable for some pupils. The suitability of a game system should therefore be considered in relation to the group, the intended session length, facilitator experience and the components of the conceptual model that the programme is intended to explore.

Potential adaptations include reducing the number of rules used, providing pre-generated or partially completed characters, using visual summaries, limiting the range of available actions and developing shorter scenarios that can reach a meaningful stopping point within a school session. Participation should not depend upon performing a character voice or improvising extended dialogue. Pupils might contribute through describing actions, making decisions, recording information, supporting another player or helping the group consider possible solutions. Alternative systems may be preferable where they provide a more accessible genre, lower cognitive demands or a structure better suited to the pupils and school context. The aim is therefore to preserve meaningful choice, collaborative storytelling and consequential participation rather than fidelity to a particular commercial game system.

### 3.4. Facilitation, Safeguarding and Suitability

The facilitator would guide the narrative, explain and adapt the rules, support participation and manage the group environment. Although this role is not equivalent to providing psychological therapy, it should not be treated as requiring no specialist knowledge or preparation. Facilitators would need sufficient familiarity with the game, confidence in managing group interaction, understanding of anxiety-related participation difficulties and knowledge of the school’s safeguarding procedures. They would also need to balance narrative structure with responsiveness, allowing pupils’ contributions to affect the story without permitting individual players to dominate or exclude others.

Psychological safety cannot be assumed simply because participation takes place through fictional characters. Research into other school-based group interventions has found that groups can provide reassurance, shared experience and a sense of belonging, but concerns about confidentiality, discomfort in front of peers and particular group dynamics can also restrict participation (McKeague et al., 2018; Soares & Woods, 2022). Group composition would therefore require careful consideration, including existing peer relationships, power differences, communication preferences and any known conflict between potential participants.

TTRPG participation may not be suitable or appealing for every young person experiencing anxiety. Improvisation, uncertainty, complex rules and performing in front of peers may themselves be anxiety-provoking. Some pupils may experience the social and cognitive demands of group gameplay as overwhelming, while others may not identify with fantasy role-play or may be uncomfortable with particular narrative themes. These difficulties may sometimes be reduced through adaptation, preparation and alternative ways of contributing, but pupils should also be able to decide that the activity is not for them without losing access to other forms of support.

Clear expectations should be established concerning respectful interaction, narrative boundaries and the limits of confidentiality within a school-based group. Participants should have accessible ways to pause, decline a particular action or step away temporarily without being penalised within the game or group. Narrative content should be developmentally appropriate and responsive to participants’ expressed boundaries. Themes involving violence, loss, threat, identity or interpersonal conflict may require particular care, even when presented fictionally.

The approach should not be designed to elicit personal disclosure. Nevertheless, pupils may connect fictional events with their own experiences or disclose concerns during or after gameplay. Facilitators should respond supportively while following existing school safeguarding and referral procedures rather than attempting to process the disclosure through the narrative. Training and guidance would therefore need to cover boundaries, signs of distress, responses to disclosure, group management and procedures for reviewing whether continued participation remains appropriate.

### 3.5. Feasibility and Implementation Support

The material requirements of a TTRPG group may be modest, but this should not be equated with the approach being low-cost, scalable or straightforward to implement. Relevant resource demands include staff preparation and delivery time, training, supervision, suitable space, coordination with existing provision and the development or adaptation of accessible gameplay materials. These requirements are currently unknown and should be measured rather than treated as established advantages.

Evidence from other school-based interventions illustrates the difficulty of fitting regular support within secondary-school systems. Withdrawal from lessons can increase anxiety about missed work, lunchtime sessions may be disrupted or rushed, and after-school delivery can reduce attendance (McKeague et al., 2018; Soares & Woods, 2022). Curriculum pressures may also alter intervention design: during the development of one school-based programme, an intended twelve-week format was reduced to six weeks to make delivery more manageable (Neill et al., 2023). Staff time, access to rooms, training, materials and the ability to provide follow-up have similarly affected whether school interventions could be delivered consistently or sustained beyond their initial implementation (McGeechan et al., 2019; McKeague et al., 2018).

Delivery by pastoral staff, learning mentors, teaching assistants or other school professionals may be possible, but it remains a proposition requiring evaluation. Future work would need to identify the minimum knowledge and preparation required, the amount of planning time involved, the forms of supervision or consultation needed and the extent to which delivery remains consistent across facilitators. Simplifying gameplay may reduce some demands, but excessive standardisation could also weaken the participant choice and co-constructed storytelling upon which the conceptual rationale depends.

Educational Psychologists may contribute through consultation rather than routine delivery. This could include supporting schools to define the intended population, consider group composition, identify barriers to participation and adapt rules, materials or narrative structures. EPs may also assist in developing facilitator guidance, establishing safeguarding and referral boundaries and evaluating whether the programme is operating in accordance with its proposed model. Their systemic position may be particularly useful in examining how timetable arrangements, staff capacity, pupil voice and wider school practices affect implementation.

These implementation requirements should be examined through the staged programme of research outlined in Section 4. The immediate question is not simply whether a school can run a Dungeons & Dragons group, but whether deliberately structured and facilitated collaborative role-play can be delivered safely and acceptably whilst preserving the components set out in the conceptual model.

## 4. Future Research Directions

Research concerning TTRPGs and psychological wellbeing remains methodologically varied, with much of the available evidence coming from small qualitative studies, adult or specialist populations, and therapeutic or community settings (Henrich & Worthington, 2023; Yuliawati et al., 2024). Research in mainstream secondary schools should therefore begin by establishing whether a deliberately structured TTRPG approach can be delivered safely and acceptably to the intended population before attempting to determine whether it reduces anxiety. This reflects the purpose of feasibility work: to establish whether an approach can be delivered, whether progression is justified, and how subsequent evaluation should proceed (Eldridge et al., 2016). A staged programme of research would allow the conceptual model to be refined while preventing preliminary findings from being interpreted as evidence of effectiveness.

### 4.1. Feasibility and Acceptability

The immediate priority should be the co-development and feasibility testing of an initial school-based model. Pupils, school staff and relevant professionals could contribute to decisions about how the activity is introduced, the accessibility of the rules, session timing, group composition, narrative boundaries and the support required by facilitators. This process should include pupils without previous TTRPG experience, rather than relying solely on existing players or young people already enthusiastic about Dungeons & Dragons.

Initial studies should examine recruitment, uptake, attendance, retention and reasons for declining or withdrawing from participation. Acceptability is a multifaceted construct encompassing participants’ cognitive and emotional responses and should not be inferred solely from behavioural indicators such as uptake, retention or withdrawal (Sekhon et al., 2017). Nor should it be inferred only from the positive accounts of pupils who complete the programme. Interviews or focus groups with participants, facilitators and, where possible, young people who decline or discontinue participation could identify elements that feel engaging, confusing, uncomfortable or stigmatising. This emphasis on pupil and staff perspectives is consistent with qualitative research into the feasibility and acceptability of school-based support (McKeague et al., 2018). Particular attention should be given to whether participants understand the purpose of the group, experience meaningful choice and feel able to influence or step back from the activity.

Feasibility evaluation should also record preparation and delivery time, facilitator training and confidence, access to suitable spaces, timetable disruption, adaptations made during delivery and the extent to which planned components are implemented consistently. Monitoring should include unintended or adverse experiences, such as distress caused by narrative content, conflict or exclusion within the group, anxiety associated with improvisation or social performance, pressure to disclose personal experiences, or difficulty managing the game’s cognitive demands. These findings would indicate whether the model requires further adaptation and whether progression to outcome evaluation is justified.

### 4.2. Mechanisms and Process Evaluation

Process evaluation should examine implementation, mechanisms of impact and context, including whether the components identified in Table 1 are experienced in the ways proposed (Moore et al., 2015). For example, research could explore whether fictional characters provide participants with a manageable degree of narrative distance, whether decisions produce a genuine sense of agency and whether the collaborative structure creates opportunities for contribution and social connection. These questions concern experiences occurring during participation and should initially be examined without assuming that they lead to changes in anxiety outside the game.

Mixed-methods designs may be particularly valuable at this stage. Session observations, facilitator records, brief participant measures and qualitative interviews could be used to examine how pupils respond to particular components and how experiences vary according to facilitator practice, group composition, previous gaming experience and adaptations to the system. This would help distinguish mechanisms that are plausibly associated with particular features of gameplay from benefits that participants attribute more generally to an enjoyable or supportive group.

Larger subsequent studies could examine whether changes in proposed proximal experiences, such as perceived safety, agency, social participation or willingness to return, are associated with later psychological or educational outcomes. Formal mediation analysis would require sufficiently powered samples and should follow, rather than precede, evidence that the programme is feasible and that the proposed processes can be measured reliably.

### 4.3. Preliminary Outcome Evaluation

If feasibility and acceptability are established, pilot controlled studies could examine preliminary outcomes and inform the design of more robust evaluations. Where the programme is explicitly intended as early anxiety support, anxiety-related functioning should be identified as a primary outcome and assessed using developmentally appropriate, validated measures. Outcomes should be measured beyond the immediate end of the programme to investigate whether any reported changes are sustained and whether experiences arising during gameplay transfer into everyday school life.

Educational and relational outcomes may also be relevant, including school belonging, engagement, classroom participation and social confidence. However, these should be selected in advance and clearly linked to the conceptual model rather than treated as interchangeable indicators of success. Attendance may be appropriate where anxiety-related attendance difficulty forms part of the participant’s presenting need, but it would be less meaningful as a general outcome for pupils whose attendance is already secure.

Controlled designs should compare the TTRPG approach with existing school support or another appropriate activity rather than assuming that change over time results from the game itself. Comparison with a collaborative non-TTRPG activity may help distinguish the contribution of role-play and co-constructed narrative from more general effects of adult attention, peer contact, novelty or participation in an enjoyable group. Studies should continue to assess negative and unintended outcomes alongside potential benefits and should report patterns of non-participation and attrition.

### 4.4. Longer-Term Implementation and Comparative Research

Questions concerning sustainability and wider implementation should be addressed only if earlier studies establish sufficient feasibility, acceptability and preliminary evidence of benefit. Multi-site research could then examine how delivery varies across schools with different staffing, resources, pupil populations and systems of pastoral support. Relevant questions would include the cost of preparation and delivery, the level of facilitator training and supervision required, the balance between adaptation and fidelity, and whether the approach can be maintained once initial research support is withdrawn.

Comparative research could also examine whether Dungeons & Dragons offers particular advantages or disadvantages relative to simpler or differently themed TTRPG systems. This may clarify whether outcomes depend upon a particular ruleset or genre, or upon broader components such as character-mediated participation, consequential choice and collaborative storytelling. Research should also investigate for whom the approach is most and least acceptable, including the influence of previous gaming experience, anxiety presentation, communication preferences and comfort with group improvisation.

This staged agenda moves from developing an acceptable and deliverable model to examining its processes, preliminary outcomes and longer-term implementation. It would allow the potential contribution of school-based TTRPG participation to be investigated without treating theoretical plausibility or positive participant experience as evidence that the approach is already an effective intervention for adolescent anxiety.

## 5. Conclusions

Adolescents experiencing emerging or subthreshold anxiety may encounter meaningful difficulties with participation, engagement and wellbeing before meeting thresholds for specialist intervention. This creates a need to investigate forms of early support that are accessible within secondary-school contexts and responsive to how young people experience and engage with provision.

This paper has considered tabletop role-playing games as one underexplored possibility. Current evidence does not establish that TTRPG participation reduces adolescent anxiety, particularly when much of the available research involves small samples and adult, therapeutic or community settings. However, findings from existing TTRPG research, broader psychological theory and young people’s experiences of school-based interventions provide a coherent basis for further investigation. The conceptual model developed in this paper connects particular features of collaborative role-play with proposed mechanisms, proximal experiences and outcomes requiring evaluation. Its central proposition is that the potential contribution of TTRPGs may lie in the interaction between co-constructed storytelling, fictional characters, meaningful choice, collaborative problem-solving and sustained social participation.

The feasibility and value of this approach will depend upon how it is designed and implemented. Participation should be voluntary, the intended population clearly defined and the game system adapted to the needs and preferences of the group. Psychological safety cannot be assumed, and careful attention will be required to facilitator preparation, group composition, narrative content, accessibility, safeguarding and the possibility that role-play may be uncomfortable or anxiety-provoking for some pupils. A school-based TTRPG approach should complement rather than replace specialist assessment or evidence-based treatment where these are required.

Research should therefore proceed in stages, beginning with co-development and feasibility and acceptability studies before progressing to process evaluation and controlled investigation of anxiety-related and educational outcomes. Such work will need to examine not only whether an approach is associated with benefit, but how it is experienced, which components appear important, what adverse or unintended effects occur and whether experiences within gameplay transfer beyond the fictional setting. The framework presented here provides a basis for determining whether, for whom and under what school conditions collaborative TTRPG participation may make a useful contribution to early anxiety support.

## Figures and Tables

**Table 1:** Proposed component–mechanism–outcome model for school-based TTRPG participation.

TTRPG Component	Proposed Mechanism	Proximal Experience During Participation	Potential Longer-Term Outcome Requiring Evaluation	Primary Basis for the Proposition
Fictional characters and co-constructed narrative	Narrative distance	Indirect exploration of emotionally relevant themes with reduced demands for personal disclosure, perceived safety, and willingness to engage	Emotional expression beyond the immediate game context	Therapeutic story-writing literature and emerging TTRPG accounts (Waters, 2004; Yuliawati et al., 2024)
Shared goals, group decisions, and collaborative problem-solving	Psychological safety and social connectedness	Positive peer interaction, group membership, and opportunities to contribute	Belonging, social participation, and emotional wellbeing	Limited TTRPG research and the broader connectedness literature (Bond et al., 2007; Abbott et al., 2022; Kilmer et al., 2024)
Meaningful choices with narrative consequences	Autonomy, competence, and agentic engagement	Ownership, acceptability, personal investment, and experience of influencing the activity	Sustained participation, and engagement with support	Self-Determination Theory and educational engagement research (Ryan & Deci, 2000; Reeve, 2013)
Character creation and experimentation with roles or behaviour	Identity exploration and self-efficacy	Exploration of possible identities, contribution to success, and experience of agency	Confidence, self-concept, coping, and transfer beyond the game	Narrative identity and self-efficacy theory, supported by qualitative TTRPG accounts (Bandura, 1997; McAdams, 2001; Yuliawati et al., 2024)
Unpredictable narrative developments, rule-governed challenges, and fictional setbacks	Cognitive flexibility and emotional regulation	Adapting plans, considering alternatives, and remaining engaged with uncertainty or disappointment	Flexible coping, tolerance of uncertainty, emotional regulation, and anxiety-related functioning	Broader psychological theory; currently a predominantly theoretical TTRPG proposition (Gross, 1998; Diamond, 2013)

Note: TTRPG = tabletop role-playing game.

## Data Availability

No new data were created or analyzed in this study. Data sharing is not applicable to this article.
